# Supplementary material for: Resolving the full spectrum of human genome variation using Linked-Reads
Source: Genome Res. 2019 Apr;29(4):635–45. doi: 10.1101/gr.234443.118 (PMC6442396; doi:10.1101/gr.234443.118)
Supplement: Supplemental Material [file supp_gr.234443.118_Supplemental_longranger-2.2.zip › longranger-2.2.tar.bz2!/lr2.2-public/lib/go/src/code.google.com/vitess/web/vtctld/shard.html]

## Shard Record


Shard Actions
more\_vert


{{actions.label(action)}}


### Keyspace/Shard

### Master Tablet

None


## Tablets (by cell)


### No tablets found.


### {{tablet.alias.cell}}-{{tablet.alias.uid}} [{{tabletType(tablet)}}]


Tablet Actions
more\_vert


{{actions.label(action)}}


**Host**
{{tablet.hostname}}:{{tablet.port\_map.vt}}

**IP Address**
{{tablet.ip}}:{{tablet.port\_map.vt}}

**Seconds Behind Master**

**Health Error**

{{link.title}}
